# Antifibrotic effects of specific targeting of the 5‐hydroxytryptamine 2B receptor (5‐HT_2B_R) in murine models and ex vivo models of scleroderma skin

**DOI:** 10.1002/art.43151

**Published:** 2025-04-17

**Authors:** Thuong Trinh‐Minh, Cuong Tran‐Manh, Andrea‐Hermina Györfi, Nicholas Dickel, Christoph Liebel, Xiang Zhou, Jiucun Wang, Meik Kunz, Helena Arozenius, Lars Pettersson, Sam Lindgren, Christina Wenglén, Jörg H. W. Distler

**Affiliations:** ^1^ Department of Rheumatology, University Hospital Düsseldorf, Heinrich Heine University, Düsseldorf, Germany; Hiller Research Center University Hospital Düsseldorf, Heinrich Heine University Düsseldorf Germany; ^2^ Chair of Medical Informatics Friedrich‐Alexander University of Erlangen‐Nürnberg Erlangen Germany; ^3^ State Key Laboratory of Genetic Engineering, School of Life Sciences and Human Phenome Institute Fudan University Shanghai China; ^4^ AnaMar AB Stockholm Sweden

## Abstract

**Objective:**

Systemic sclerosis (SSc) is a connective tissue disease with fibrotic remodeling of the skin and various internal organs. SSc is associated with the highest case‐specific mortality of all rheumatic autoimmune diseases with limited antifibrotic treatment options. Here, we evaluated the therapeutic effects of the highly selective 5‐hydroxytryptamine 2B receptor (5‐HT_2B_R) inhibitor AM1476.

**Methods:**

The antifibrotic effects of AM1476 were evaluated in the mouse models of bleomycin‐induced pulmonary fibrosis in Tsk‐1 mice and in mice with sclerodermatous chronic graft‐versus‐host disease. For further validation, the antifibrotic effects of AM1476 were analyzed in precision cut skin (PCS) slices from patients with SSc.

**Results:**

AM1476 demonstrated high selectivity for 5‐HT_2B_R over more than 200 other receptors, including other 5‐HT receptors in vitro. AM1476 reduced accumulation of hydroxyproline and fibrotic tissue remodeling of skin and/or lungs in all three mouse models at well‐tolerated doses with a comparable efficacy to that of nintedanib. In PCS of SSc skin, treatment with AM1476 reduced the expression of SSc‐specific signature genes. AM1476 demonstrated more pronounced regulation of terms related to fibroblast activation and fibrotic remodeling than mycophenolate mofetil.

**Conclusion:**

We describe AM1476 as a highly selective inhibitor of 5‐HT_2B_R. Treatment with AM1476 ameliorated fibrosis in three mouse models of SSc and normalized the expression of fibrosis‐related genes directly in SSc skin. Because AM1476 also demonstrated good tolerability in a phase 1 trial, further clinical trials with AM1476 are currently in the planning stage.

## INTRODUCTION

Fibrosis describes the excessive deposition of extracellular matrix in a tissue.^1^ Aberrant fibrotic remodeling commonly induces organ dysfunction or failure. Fibrosis is one of today's major health care challenges. Fibrotic tissue remodeling accounts for up to 40% of the deaths in industrialized countries and results in socioeconomic costs of dozens of billions of euros per year to date.^2^ Moreover, the global incidence of fibrosis and the associated health care burden are further increasing.^3^ Systemic sclerosis (SSc) is an idiopathic systemic fibrotic disease of the skin and multiple internal organs and is associated with the highest case‐specific mortality of all autoimmune rheumatic diseases, with half of the patients dying as a direct consequence of the disease.^4^ Despite considerable efforts, only a few antifibrotic therapies are approved for clinical use, generating a great need for novel effective antifibrotic therapies.

Myofibroblasts are activated fibroblasts that express contractile proteins and release abundant amounts of collagen and other components of the extracellular matrix. Although these cells are considered as core effector cells of all fibrotic diseases, the mechanisms that lead to their accumulation in fibrotic tissues remain incompletely understood. However, one recently identified mechanism is the activation of the 5‐hydroxytryptamine 2B receptor (5‐HT_2B_R) by serotonin released from platelets at sites of vascular damage in SSc.^5^ In addition to fibroblast modulation, 5‐HT regulates macrophage plasticity, and activation of the 5‐HT_2B_R promotes profibrotic alternatively activated M2 macrophages (AAMs), a rich source of transforming growth factor β (TGFβ).^6^ However, clinical translation of these findings has been hindered by the lack of highly selective, peripherally acting inhibitors of 5‐HT_2B_R with sufficient pharmacokinetics for clinical use.

We describe here the development and antifibrotic effects of a novel 5‐HT_2B_R inhibitor intended for oral treatment in humans. AM1476 (AnaMar AB; Pettersson, L. PCT appl. WO2016/207231) is an orally bioavailable small molecule targeting the peripheral 5‐HT_2B_R. AM1476 is a potent and competitive 5‐HT_2B_R antagonist devoid of agonistic activity and highly selective for the 5‐HT_2B_R. AM1476 has a favorable pharmacokinetic profile and has no preference to distribute to the brain. AM1476 demonstrated antifibrotic effects in different murine models and ameliorated the SSc‐specific gene signature in an ex vivo model of SSc skin.

## MATERIALS AND METHODS

### Treatment

AM1476 was dissolved in sterile distilled water for the ex vivo and in vivo experiments. A concentration of 30 μ*M* was applied for the ex vivo experiment. For the in vivo experiments, 1, 3, or 10 mg/kg was administered orally once or twice daily.

### Receptor binding and functionality profile of the 5‐HT_2B_R antagonist AM1476

The binding profile of AM1476 was evaluated for binding to human 5‐HT_2A_R, 5‐HT_2B_R, and 5‐HT_2C_R in radioligand binding assays using Chinese hamster ovary (CHO‐K1) cells overexpressing human receptors (in vitro assays 271650, 271700, and 271800; Eurofins Panlabs Discovery). AM1476 was evaluated at 0.1, 1, 10, 100, 1,000, and 10,000 n*M* (5‐HT_2B_R) or at 1, 10, 100, 1,000, and 10,000 n*M* (5‐HT_2A_R and 5‐HT_2C_R). Human 5‐HT_2A_R and 5‐HT_2B_R functionality was measured with homogeneous time‐resolved fluorescence quantification of IP1 accumulation in CHO‐K1 cells (in vitro assays 355250 and 355260). Compounds creating a ≥50% inhibition of 5‐HT (5 n*M*) induced fluorescence response specified receptor antagonist activity. 5‐HT_2C_R functionality was assessed with [35S] GTPγS for quantification of bound GTPγS. Compounds creating a ≥50% inhibition of 5‐HT (100 n*M*) induced [35S] GTPγS binding response specified receptor antagonist activity (in vitro assay 355200). AM1476 was evaluated at 0.1, 0.3, 1, 3, 10, 30, and 300 n*M* (5‐HT_2B_R) or 1, 10, 100, 1,000, and 10,000 n*M* (5‐HT_2A_R and 5‐HT_2C_R). A nonlinear least squares regression analysis was used to determine half‐maximal inhibitory concentration (IC_50_) values. Mouse 5‐HT_2B_R functionality was measured in a calcium influx assay (Charles River) in CHO‐KI cells expressing the mouse 5‐HT_2B_R using a calcium sensitive dye and a fluorescence imaging plate reader (FLIPRTETRA) instrument. AM1476 was evaluated at 0.3, 1, 3, 10, 30, 100, 300, and 1,000 n*M*.

Effects were considered significant if AM1476's mean value was three or more SDs above the vehicle control mean in the agonist assay or three or more SDs below the positive control agonist (5‐n*M* α‐methylserotonin) mean in the antagonist assay. AM1476 was screened for selectivity against approximately 200 targets using a Eurofins Panlabs Spectrum screen panel including enzymatic and receptor binding assays. Targets with potential peripheral effects to which AM1476 displayed a hit, defined as ≥50% inhibition at 10 μ*M*, were further evaluated at a lower concentration and/or in functionality assays at Eurofins Panlabs.

### Patients

Ten patients with a diagnosis of SSc according to the 1980 American College of Rheumatology (ACR) criteria for SSc^7^ and the 2013 EULAR/ACR classification criteria^8^ provided informed written consent to participate in the study and were included. Only patients over 18 years of age were included in the study. One patient with SSc, who developed symptoms of myositis on clinical follow‐up and was diagnosed with an overlap syndrome of SSc and dermatomyositis, was excluded from the analysis. The study was approved by the ethical committee of Friedrich‐Alexander University Erlangen‐Nürnberg and of the Heinrich Heine University Düsseldorf (applications 30‐19B, 98‐18B, and 2022‐2158). Human studies were performed in compliance with the relevant ethical regulations. Patient demographics and clinical characteristics are provided in Supplementary Table S1.

### Mouse models

#### Experimental sclerodermatous chronic graft‐versus‐host disease

The B10.D2→BALB/c (H‐2d) minor histocompatibility antigen–mismatched model was performed as described.^9^, ^10^ Briefly, female BALB/c (H‐2d) mice were purchased from Janvier. Male B10.D2 (H‐2d) mice were initially purchased from Jackson Laboratory. All mice were maintained in specific pathogen‐free conditions with sterile pellet food and water and a normal day–night cycle. For isolation of unfractionated bone marrow cells, tibial and femoral bones were prepared under sterile conditions. Phosphate buffered saline (PBS) was used to flush bone marrow cells from bone marrow cavities. Subsequently, bone marrow cells were filtered through 70‐μm nylon meshes (BD Biosciences), followed by erythrocyte hemolysis. The remaining bone marrow cells were kept on ice until transplantation. Transplantation of bone marrow cells and splenocytes was performed as follows: Recipient mice (BALB/c [H‐2d]) eight weeks of age received total body irradiation with 750 cGy. Six hours after irradiation, all BALB/c (H‐2d) recipients received bone marrow from either BALB/c (H‐2d) in a syngeneic transplantation manner or B10.D2 (H‐2d) in an allogeneic transplantation manner. For transplantation, 5 × 10^6^ splenocytes and 2 × 10^6^ bone marrow cells from donor mice were resuspended in 0.2 mL of PBS and injected via tail veins.^9^, ^10^ To reflect the clinical situation with treatment initiation upon clinical signs of chronic graft‐versus‐host disease (cGvHD), treatment was started 21 days after bone marrow transplantation (BMT) and thus after the first clinically detectable manifestations of cGvHD in allogeneically transplanted mice. The outcome of treatment was analyzed seven weeks after transplantation. Eight mice per group were analyzed.

#### Tsk‐1 mouse model

In the murine Tsk‐1 model of SSc, a dominant mutation in the fibrillin 1 gene leads to an SSc‐like disease, with minor inflammatory infiltrates, autoantibody production, and fibrosis of the skin. This model reflects the later stages of SSc characterized by noninflammatory self‐sustaining fibrosis. Eight to 12 mice per group were analyzed. Treatment was initiated at an age of five weeks, and the outcome was analyzed at an age of 10 weeks.^11^

#### Bleomycin‐induced pulmonary fibrosis

Pulmonary fibrosis was induced by a single intratracheal instillation of bleomycin (25 μg in 50 μL of 0.9% NaCl).^12^ Treatment was initiated at two weeks after the first injection of bleomycin. AM1476 was administered orally at 1, 3, or 10 mg/kg once daily. Solvent (double‐distilled water) was used as a control, and nintedanib at 30 mg/kg orally every day (qd) was included as a comparator. The outcome was analyzed at two and four weeks after the first injection of bleomycin. Mice injected with 0.9% NaCl, the vehicle of bleomycin, served as nonfibrotic controls. Eight mice per group were analyzed.

#### General monitoring of mice

Mice were monitored clinically on a daily basis for behavior, activity, texture of the fur, weight, and consistency of the stool.^13^ After the mice were killed, a gross macroscopic evaluation of the lungs and the skin was performed.

#### Clinical scoring of cutaneous cGvHD

Recipient mice were clinically monitored on a daily basis from the day of transplantation to the indicated days after transplantation to determine the incidence and severity of cutaneous cGvHD as well as mobility, diarrhea, and weight loss.^10^, ^14^ The following scoring system for cutaneous cGvHD was used: healthy appearance = 0; skin lesions with alopecia <1 cm^2^ in area = 1; skin lesions with alopecia 1 to 2 cm^2^ in area = 2; and skin lesions with alopecia >2 cm^2^ in area = 3. Incidence was expressed as the percentage of mice that showed clinical manifestations.

### Histologic evaluation of dermal and pulmonary fibrosis

Skin samples were fixed in 4% formalin for six hours and embedded in paraffin. Five‐micrometer sections were cut and stained with hematoxylin and eosin (H&E). The dermal thickness was quantified on H&E‐stained sections using images captured with a light microscope (Nikon eclipse 80i) at 100‐fold magnification by manually measuring the distance between the epidermal‐dermal junction and the dermal‐subcutaneous fat junction at four sites per mouse.^15^, ^16^ For lung samples, the whole left lung was fixed in 4% formalin for six hours and embedded in paraffin. Five‐micrometer sections were cut and stained with trichrome and sirius red. Histologic readouts were evaluation of the fibrotic area as the percentage of the total lung area in sirius red–stained sections (two sections per mouse) and quantification of pulmonary changes using the Ashcroft score (four sections per mouse).^17^, ^18^

### Hydroxyproline assay

The amount of collagen protein in skin and lung samples was determined by hydroxyproline assay.^19^, ^20^ After digestion in 6 *M* HCl for three hours at 120°C, the pH of the samples was adjusted to a pH range of 6–7 with 6 *M* NaOH. Afterward, 0.06 *M* chloramine T was added to each sample and incubated for 20 minutes at room temperature. Next, 3.15 *M* perchloric acid and 20% *p*‐dimethylaminobenzaldehyde were added, and samples were incubated for an additional 20 minutes at 60°C. The absorbance was determined at 557 nm with a Spectra MAX 190 microplate spectrophotometer. Absolute values were determined using a standard curved generated with type I collagen (Sigma‐Aldrich).

### Detection of myofibroblasts

Myofibroblasts are characterized by the expression of α‐smooth muscle actin (α‐SMA). Fibroblasts positive for α‐SMA were detected by immunohistochemistry staining with monoclonal anti–α‐SMA antibodies (clone 1A4, Sigma‐Aldrich). The expression was visualized with horseradish peroxidase–labeled secondary antibodies and 3,3‐diaminobenzidine tetra hydrochloride (Sigma‐Aldrich). Monoclonal mouse IgG antibodies (Calbiochem) were used as controls. Images of sections stained for α‐SMA were captured on a light microscope (Nikon eclipse 80i). The myofibroblast number was manually evaluated at four different areas at 200‐fold magnification. Myofibroblasts were defined as α‐SMA positive, single, and spindle‐shaped cells in the dermis.^5^, ^21^

### Immunofluorescence staining

Immunofluorescence staining was performed as described previously.^22^ AAMs are defined as cells positively stained for anti‐cMAF antibody (PA5‐23179, Thermo Scientific, 1:100 dilution), anti‐arginase antibody (ab60176, Abcam, 1:100 dilution), and anti‐F4/80 antibody (MCA497G, Bio‐rad, 1:100 dilution). Donkey anti‐rat Alexa Fluor 488, donkey anti‐goat Alexa Fluor 555, and donkey anti‐rabbit Alexa Fluor 647 (Life Technologies) were used as secondary antibodies for AAM staining. Counter staining was performed with the DNA dye DAPI (Santa Cruz Biotechnology). The analysis of AAM cells was performed by an experienced reviewer in a blinded manner in three sections per sample.

The staining of skin sections was performed by using the antibodies against CCL2 (LS‐C169178‐100, LSBio, 1:100 dilution), CCL5 (MAB478‐100, Biotechne, 1:100 dilution), CCL18 (22303‐1‐AP, Proteintech, 1:100 dilution), PAI‐1 (66261‐1‐Ig, Proteintech, 1:100 dilution), P‐STAT3 (MA5‐15193, Thermo Scientific, 1:100 dilution), MAOB (12602‐1‐AP, Proteintech, 1:100 dilution), or SLC6A4 (LS‐C154958‐100, LSBio, 1:100 dilution). Donkey anti‐mouse or anti‐rabbit Alexa Fluor 488 was used as secondary antibodies. Counter staining was performed with the DNA dye DAPI (Santa Cruz Biotechnology). The analysis of target average intensity was performed by an experienced researcher in a blinded manner in two sections per sample.

### RNA sequencing and bioinformatics analysis of murine and human skin

Total RNA from the cGvHD mouse model and from human precision cut skin (PCS) slices was extracted using the Qiagen total RNA kit according to the instructions of the manufacturer. RNA sequencing (RNASeq) was performed by Novogene on an Illumina NovaSeq platform using a paired‐end 150‐bp sequencing strategy. Raw paired‐end reads were mapped to the reference genome of *Homo sapiens* (GRCh38) and *Mus musculus* (GRCm38, mm10) using STAR aligner (version 2.0.2). The sorted bam files were processed by featureCounts to obtain a count matrix. Principal component analysis was performed using the ggplot2 package (version 3.4.0) to determine the potential outliers among samples in each batch. After quality control, a differential gene expression analysis was performed using edgeR (version 3.38.4), in which a normalization method Trimmed Mean of M‐values was applied. A threshold of adjusted *P* ≤ 0.05 and |log_2_ fold change (log_2_FC)| ≥ 1 was used to identify the statistically significantly differentially expressed genes (DEGs). The results were visualized as a volcano plot using the ggplot2 package (version 3.4.0) according to the log_2_FC and –log10 adjusted *P* value of DEGs. Hierarchical cluster analyses were performed using the pheatmap package (version 1.0.12). A functional overrepresentation analysis was performed using clusterProfiler (version 4.4.4), and results were depicted in a dot plot. A gene set enrichment analysis (GSEA) on normalized gene counts was performed using the fgsea package (version 1.22.0), in which the collected gene sets of c2‐KEGG from the Molecular Signatures Database (MSigDB) were used. Analysis was performed in R (version 4.2.1).

### Pharmacokinetic study of AM1476

A pharmacokinetic study of AM1476 was performed using the cGvHD model described previously. After repeated oral administration for 28 days, whole blood was collected from each treatment group 15 minutes, 30 minutes, and 1, 2, 4, 6, 8, and 24 hours after a final morning dose (two animals were used for each time point), and plasma was separated from blood within 30 minutes. The plasma concentration of AM1476 was measured by a Waters TQ‐S triple quadrupole Mass Spectrometer system coupled with Acquity UPLC (Admescope).

### PCS slices of SSc skin

Freshly taken skin punches (diameter of 3 mm) of 10 patients with SSc were embedded in 3% low melting agarose (#16520100, Thermo Fisher Scientific). Cylindrical cores of solidified agarose containing the skin punch were generated, and consecutive PCS slices with a thickness of 230 μm containing epidermis, dermis, and hypodermis layers were generated using an Alabama R&D Tissue Slicer (Alabama Research and Development). PCS slices were subsequently transferred into 24‐well plates filled with prewarmed minimum essential media (Life Technologies) containing 11.34 m*M* glucose (Komtur Pharmaceuticals), 26 m*M* NaHCO_3_ (Sigma‐Aldrich), 25 m*M* HEPES, 1 m*M* sodium pyruvate, 2 m*M* GlutaMax (all Gibco), penicillin/streptomycin, and amphotericin (both Sigma‐Aldrich) at 37°C, 5% CO_2_, and atmospheric O_2_. After 12 hours, PCS slices were incubated with AM1476 (30 μ*M*), mycophenolate mofetil (1 μ*M*), or vehicle (medium) for 48 hours. Afterward, PCS slices were collected in RNAlater (#AM7020, Thermo Fisher) and stored at −80°C until RNA isolation was performed.

### Statistics

All non–RNASeq data are presented as mean ± SEM, with individual values displayed as dots. Differences between the groups were tested for their statistical significance by one‐way analysis of variance. *P* values less than 0.05 were considered significant.

## RESULTS

### Characterization of AM1476 as a selective inhibitor of 5‐HT_2B_R

**Figure 1:**
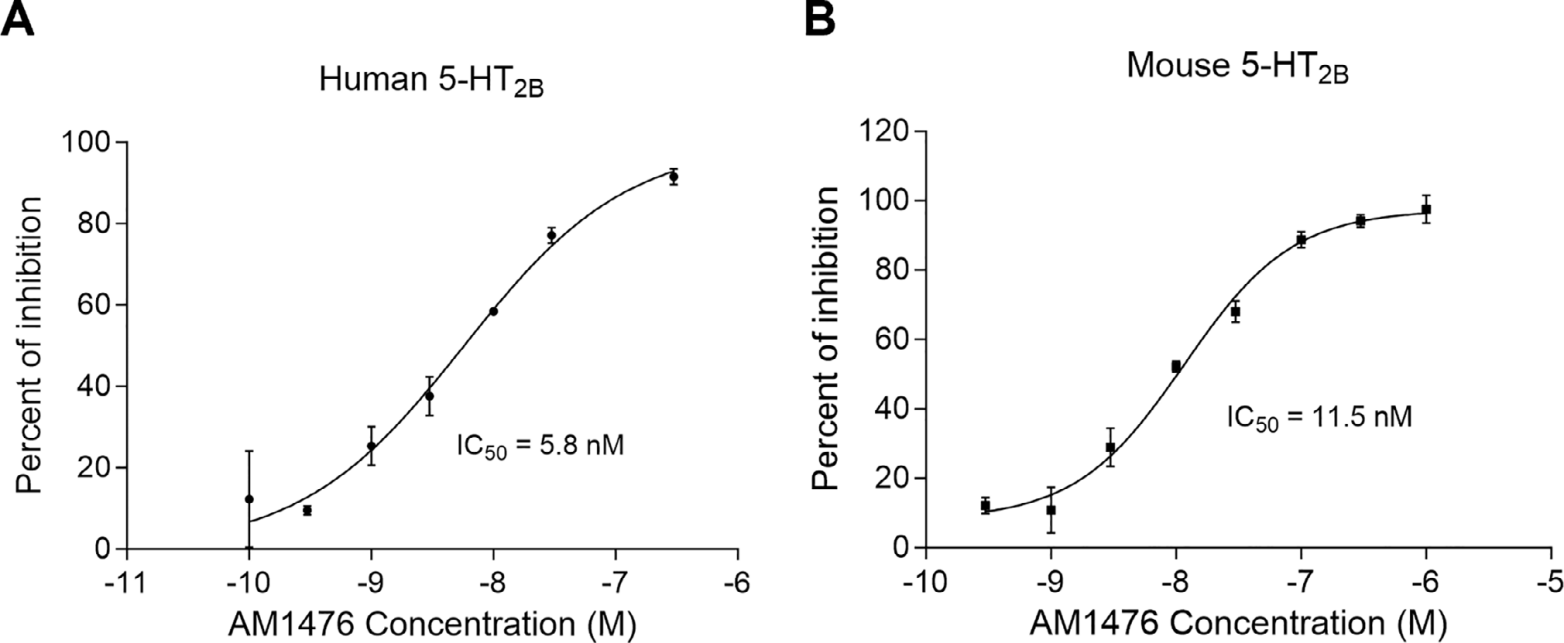
Inhibition curves for AM1476 in (A) human and (B) mouse 5‐HT_2B_R functionality assays. 5‐HT_2B_R, 5‐hydroxytryptamine 2B receptor; IC_50_, inhibitory concentration.

The 5‐HT_2_ receptor binding affinity and functionality for AM1476 AnaMar AB; PCT appl. WO2016/207231) was evaluated in vitro by Eurofins Panlabs using CHO‐K1 cells that stably express human recombinant 5‐HT_2A_R, 5‐HT_2B_R, or 5‐HT_2C_R. Receptor studies showed that AM1476 binds to the human 5‐HT_2B_R with an inhibition constant (K_i_) of 6.0 n*M*, (Ki of 800 n*M* for 5‐HT_2A_R and 60 n*M* for 5‐HT_2C_R). AM1476 strongly antagonizes the 5‐HT_2B_R with a half‐maximal IC_50_ of 5.8 n*M* (Figure 1A) but showed only low activity at the 5‐HT_2A_R (IC_50_ > 10,000 n*M*) and 5‐HT_2C_R (IC_50_ = 3100 n*M*) (Supplementary Table S2). The AM1476 IC_50_ value to mouse 5‐HT_2B_R was 11.5 n*M* (Figure 1B). Testing of approximately 200 other targets, including several members of the 5‐HT receptor family, demonstrated no binding to the majority of targets (defined as <50% inhibition at 10 μ*M*). Targets with potential peripheral effects to which AM1476 displayed a hit (defined as ≥50% inhibition at 10 μ*M*) were further evaluated at a lower concentration (Supplementary Table S3) or in functionality assays (Supplementary Table S4). No interactions at clinically relevant concentrations were detected, thereby confirming the high selectivity of AM1476 to the 5‐HT_2B_R (Supplementary Tables S3 and S4).

### Inhibition of 5‐HT_2B_R ameliorates dermal fibrosis in murine cGvHD and tight skin models

**Figure 2:**
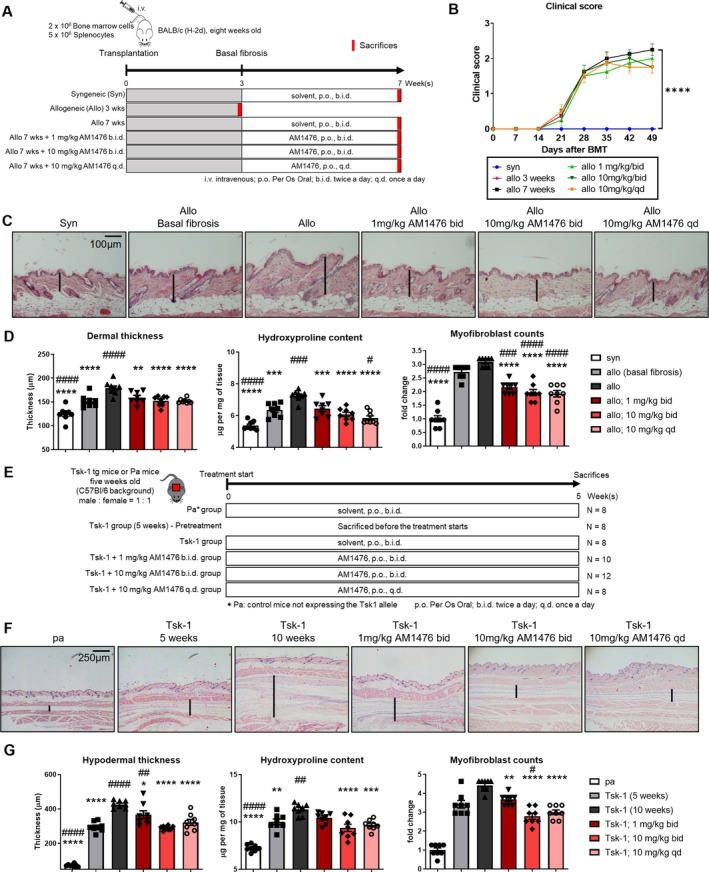
AM1476 ameliorates fibrosis in experimental cGvHD and Tsk‐1‐mice. (A–D) cGvHD‐induced dermal fibrosis. (A) Schematic illustration of the experimental design of cGvHD‐induced dermal fibrosis. (B) Effects of AM1476 on clinical cutaneous scores in cutaneous cGvHD. (C) Representative hematoxylin and eosin staining of cGvHD‐induced dermal fibrosis. (D) Quantification of dermal thickness, hydroxyproline content, and myofibroblast counts. (E–G) Skin fibrosis in Tsk‐1 mice. (E) Schematic illustration of the experimental outline. (F) Representative hematoxylin and eosin staining. (G) Quantification of hypodermal thickness, hydroxyproline content, and myofibroblast counts. All data are presented as mean ± SEM, with individual values displayed as column plus dots. Differences between the groups were tested for their statistical significance by one‐way analysis of variance with Dunnett's multiple comparison. Adjusted *P* values <0.05 were considered significant. Adjusted *P* values are expressed as follows: *0.05 > *P* > 0.01; **0.01 > *P* > 0.001; ***0.001 > *P* > 0.0001; ****P < 0.0001 as compared to fibrotic control mice (allo 7 weeks or Tsk‐1 10 weeks). Adjusted *P* values are expressed as follows: ^#^0.05 > *P* > 0.01; ^##^0.01 > *P* > 0.001; ^###^0.001 > *P* > 0.0001; ^####^ *P* < 0.0001 as compared to baseline fibrotic control mice (allo 3 weeks or Tsk‐1 5 weeks). allo, allogeneic; BMT, bone marrow transplantation; cGvHD, chronic graft‐versus‐host disease; syn, syngeneic.

We next aimed to evaluate the effects of AM1476 in mouse models of SSc (Figure 2A). We chose the cGvHD model because of its rapidly progressive and systemic fibrotic remodeling, which includes the skin and lungs as major tissues evaluated in clinical trials of SSc. Allogeneic BMT induced progressive weight loss, with a significantly lower mean body weight in allogeneically transplanted, vehicle‐treated mice compared to syngeneic controls 49 days after BMT (Supplementary Figure 1). The composite score of cutaneous cGvHD progressively increased in vehicle‐treated, allogeneically transplanted mice after BMT compared to syngeneic controls (Figure 2B). Moreover, allogeneic transplantation induced prominent skin fibrosis with increased dermal thickness, up‐regulated the hydroxyproline content, and induced the accumulation of myofibroblasts (Figure 2C and D). Dermal thickness, hydroxyproline content, and myofibroblasts numbers increased over time, with higher levels at seven weeks after BMT as compared to three weeks after BMT (Figure 2D).

Treatment with all three doses of AM1476 was well tolerated without obvious signs of toxicity on clinical examination, on gross necropsy, or on histology at any dose. Treatment with AM1476 started at day 21 after BMT demonstrated antifibrotic effects on cGvHD‐induced skin fibrosis and reduced dermal thickening, accumulation of hydroxyproline, and myofibroblast differentiation as compared to vehicle‐treated mice observed for seven weeks after BMT (Figure 2C). The antifibrotic effects of doses of 10 mg/kg twice a day (bid) and 10 mg/kg qd were comparable to each other, and both were more pronounced than with 1 mg/kg bid. Myofibroblast counts of mice treated with all three doses were significantly lower than that of mice killed three weeks after BMT (Figure 2D). A significant reduction compared to mice killed after three weeks was also observed for the hydroxyproline content in mice treated with doses of 10 mg/kg qd (Figure 2D).

In the murine Tsk‐1 model of SSc, a dominant mutation in the fibrillin 1 gene leads to skin fibrosis. This model resembles features of later stages of SSc without major inflammatory infiltrates in the skin, where fibrotic remodeling is mainly driven by endogenously active fibroblasts. Tsk‐1 mice do not show major changes in the thickness of the dermal layer, but extracellular matrix (ECM) accumulates in the hypodermis. Tsk‐1 mice developed prominent skin fibrosis with hypodermal thickening, myofibroblast differentiation, and increased hydroxyproline content that progressed between ages 5 and 10 weeks. All three doses of AM1476 reduced hypodermal thickening, myofibroblast counts, and hydroxyproline content compared to vehicle‐treated 10‐week‐old Tsk‐1 mice (Figure 2E–G). The hypodermal thickness and the hydroxyproline content of mice treated with 10 mg/kg qd or treated with 10 mg/kg bid were comparable to that of five‐week‐old Tsk‐1 mice (pretreatment level) (Figure 2F and G). Plasma concentrations of AM1476 after 28 days of repeated oral once daily administration in the cGvHD mice were measured. The results show that the level of systemic exposure of AM1476 exceeded the IC_50_ of mouse 5‐HT_2B_R antagonistic activity (Supplementary Figure 2).

### AM1476 alleviates pulmonary fibrosis induced by cGvHD and bleomycin

**Figure 3:**
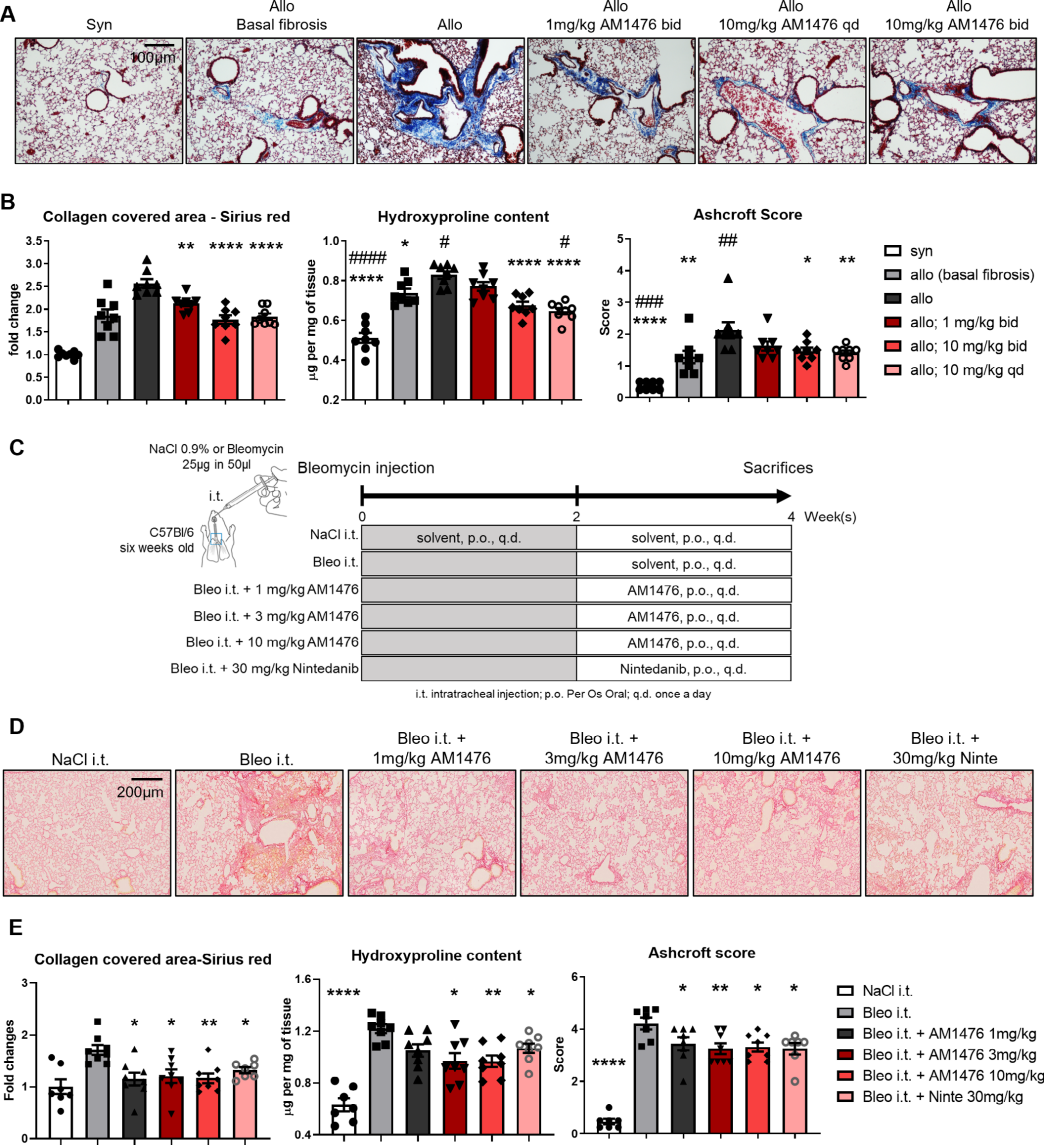
Inhibition of 5‐HT_2B_R by AM1476 ameliorates cGvHD‐ and bleomycin‐induced pulmonary fibrosis. (A and B) cGvHD‐induced pulmonary fibrosis. (A) Representative trichrome staining in lungs from cGvHD‐induced dermal fibrosis experiment and (B) quantifications of collagen‐covered area (sirius red staining), hydroxyproline content, and Ashcroft score. (C–E) Bleomycin‐induced pulmonary fibrosis: (C) Schematic illustration of the experimental design of bleomycin‐induced pulmonary fibrosis. (D) Representative sirius red staining from lung sections. (E) Quantification of collagen‐covered area (sirius red staining), hydroxyproline content, and Ashcroft scores. All data are presented as mean ± SEM, with individual values displayed as column plus dots. Differences between the groups were tested for their statistical significance by one‐way analysis of variance with Dunnett's multiple comparison. Adjusted *P* values are expressed as follows: *0.05 > *P* > 0.01; **0.01 > *P* > 0.001; ***0.001 > *P* > 0.0001; ****P < 0.0001 as compared to fibrotic control mice (allo 7 weeks or Bleo i.t.). Adjusted *P* values are expressed as follows: ^#^0.05 > *P* > 0.01; ^##^0.01 > *P* > 0.001; ^###^0.001 > *P* > 0.0001; ^####^ *P* < 0.0001 as compared to baseline fibrotic control mice (allo 3 weeks). 5‐HT_2B_R, 5‐hydroxytryptamine 2B receptor; allo, allogeneic; Bleo i.t., bleomycin intratracheal injection; cGvHD, chronic graft‐versus‐host disease; syn, syngeneic. Color figure can be viewed in the online issue, which is available at http://onlinelibrary.wiley.com/doi/10.1002/art.43151/abstract.

Allogeneic BMT induced moderate pulmonary fibrosis, with increases in Ashcroft scores, in the collagen‐covered lung area and in the hydroxyproline content (Figure 3A and B and Supplementary Figure 3). Treatment with AM1476 at all three doses reduced the Ashcroft scores, the collagen‐covered lung area, and the hydroxyproline content as compared to vehicle‐treated mice observed for seven weeks after BMT (Figure 3A and B). Treatment with AM1476 in doses of 10 mg/kg qd reduced the hydroxyproline levels to below those of mice killed three weeks after BMT, indicative of reversion of collagen accumulation.

We chose bleomycin‐induced pulmonary fibrosis as a well‐established model of rapidly progressive and severe pulmonary fibrosis, in contrast to the mild‐to‐moderate changes in the lungs of cGvHD mice. Bleomycin‐challenged mice developed prominent pulmonary fibrosis, with increased Ashcroft scores, increases in the collagen‐covered area, and increased hydroxyproline content (Figure 3C–E). All doses of AM1476 demonstrated antifibrotic effects, with decreases in Ashcroft scores, the collagen‐covered area, and hydroxyproline content as compared to vehicle‐treated, bleomycin‐challenged mice (Figure 3D and E). The antifibrotic effects of AM1476 were comparable to those of nintedanib, a tyrosine kinase inhibitor approved for the treatment of progressive pulmonary fibrosis, across the different readouts.

### Inhibition of 5‐HT_2B_R regulates core pathways of fibrosis in murine cGvHD

**Figure 4:**
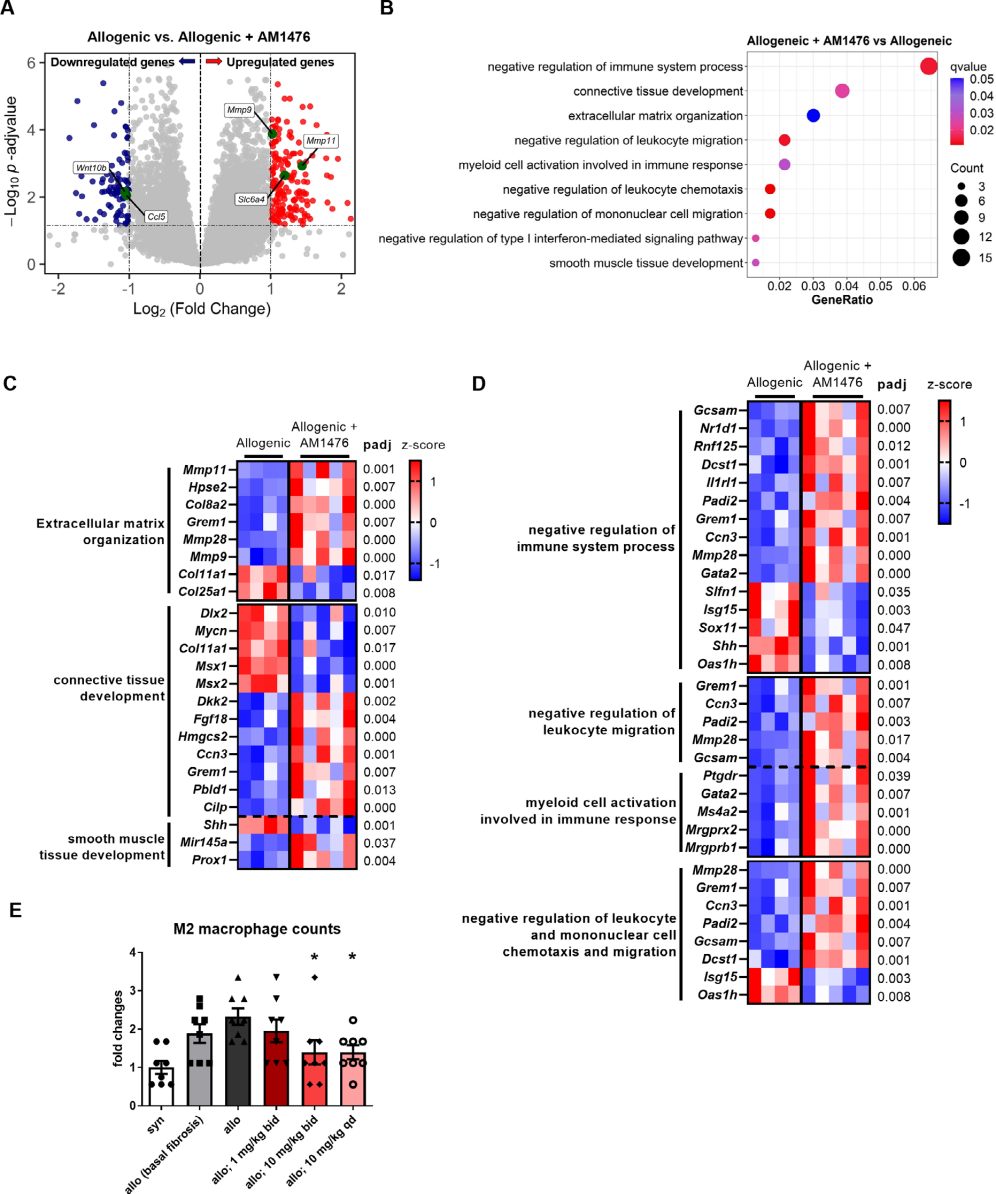
AM1476 ameliorates profibrotic transcriptional programs and alternative macrophage activation in cGvHD skin. (A) Volcano plot of the comparison of vehicle‐treated allogeneic mice (n = 4) with allogeneic mice treated with AM1476 at 10 mg/kg once daily (n = 5). (B) Functional overrepresentation analysis of AM1476‐allo‐DEGs based on the GO‐BP database showing deregulation of several gene sets related to extracellular matrix remodeling and activation of innate immunity upon treatment with AM1476. (C) Heatmap showing significant signature genes of GO‐BP enrichment terms related to fibrosis and tissue remodeling. (D) Heatmap showing significant signature genes of GO‐BP enrichment terms related to immune response. (E) Bar graphs with dots show quantification of the number of activated M2 macrophages in allo mice treated with different concentrations of AM1476. All data are presented as mean ± SEM, with individual values displayed as column plus dots. Differences between the groups were tested for their statistical significance by one‐way analysis of variance with Dunnett's multiple comparison. Adjusted *P* values <0.05 were considered significant. Adjusted *P* values are expressed as follows: *0.05 > *P* > 0.01; **0.01 > *P* > 0.001; ***0.001 > *P* > 0.0001; ****P < 0.0001 as compared to fibrotic control mice. allo, allogeneic; cGvHD, chronic graft‐versus‐host disease; DEGs, differentially expressed genes; GO‐BP, Gene Ontology Biological Process. Color figure can be viewed in the online issue, which is available at http://onlinelibrary.wiley.com/doi/10.1002/art.43151/abstract.

To obtain insights into the molecular effects of AM1476, we performed RNASeq. The comparison of samples from AM1476‐treated mice undergoing allogeneic transplantation and vehicle‐treated mice undergoing allogeneic transplantation yielded 260 DEGs, including 171 up‐regulated genes and 89 down‐regulated genes (adjusted *P* ≤ 0.05, |log_2_FC| ≥ 1) (Figure 4A).

Functional enrichment via Gene Ontology based on the Biological Process database demonstrated AM1476‐induced changes in several processes related to tissue remodeling, such as connective tissue development, extracellular matrix organization, and smooth muscle tissue development, or processes involved in inflammation, such as negative regulation of immune system process, negative regulation of leukocyte migration, myeloid cell activation involved in immune response, negative regulation of leukocyte chemotaxis, negative regulation of mononuclear cell migration, and negative regulation of the type I interferon–mediated signaling pathway (Figures 4B). We found several well‐known fibrotic genes to be down‐regulated, including *Shh*, *Col25a1*, and *Col11a1*, as well as many antifibrotic genes to be up‐regulated, including *Dkk2*, *Ccn3*, *Fgf18*, *Hpse2*, *Cilp*, and *Prox1* (Figure 4C). Moreover, many genes involved in inflammatory processes are found significantly regulated by AM1476, such as *Nr1d1*, *Mmp28*, *Sox11*, *Ccn3*, *Dcst1*, and *Isg15* (Figure 4D). AM1476 may also inhibit accumulation of AAMs. Quantifications of AAM counts in the skin also demonstrated reduction of AAM accumulation for all three doses, with statistically significant differences for 10 mg/kg qd and 10 mg/kg bid (Figure 4E). This effect was not a mere reflection of reduced inflammation upon AM1476 treatment because none of the concentrations of AM1476 reduced the number of CD45^+^ leukocytes (Supplementary Figure 4).

### Evaluation of the effects of AM1476 in PCS slices of SSc skin

**Figure 5:**
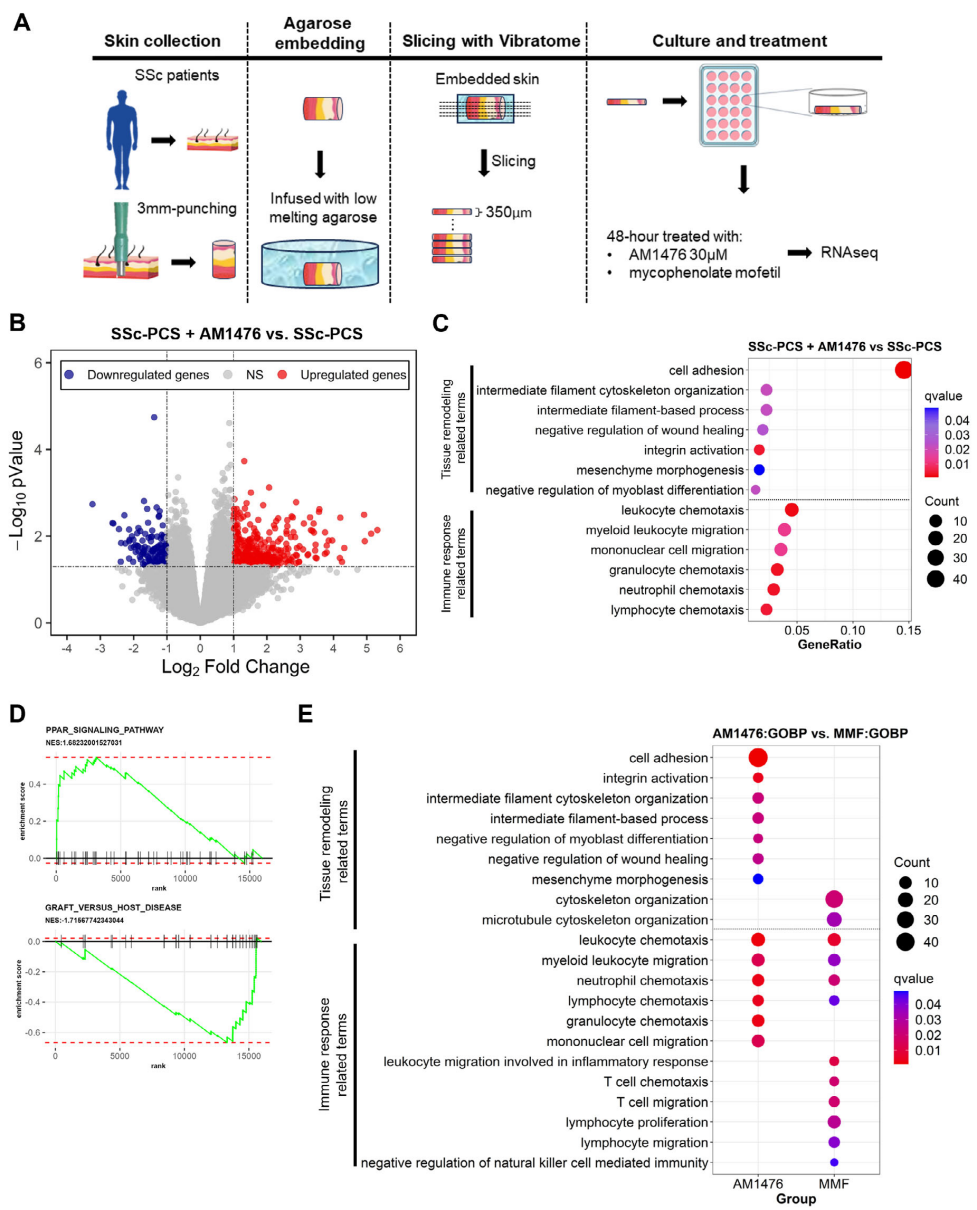
Inhibition of 5‐HT_2B_R by AM1476 modulates inflammation and fibrosis‐related transcriptional responses in SSc skin. (A) Schematic visualization of the experiment. (B) Volcano plot illustrating AM1476‐DEGs (down‐regulated genes in dark blue and up‐regulated genes in red) representing 5‐HT_2B_R inhibitor (AM1476, 30 μ*M*)–treated SSc‐PCS compared to control SSc‐PCS treated with vehicle. (C) Bubble plot of the functional overrepresentation analysis of AM1476‐DEGs showing terms related to tissue remodeling and immune response. (D) GSEA of the AM1476‐DEGs showing enrichment of the gene set “PPAR_signaling_pathway” and de‐enrichment of the gene set “graft_versus_host_disease.” (E) Bubble plot presenting the comparison of the functional overrepresentation analysis between AM1476:GOBP and MMF:GOBP showing terms related to tissue remodeling and immune response. 5‐HT_2B_R, 5‐hydroxytryptamine 2B receptor; DEGs, differentially expressed genes; GOBP, Gene Ontology Biological Process; GSEA, gene set enrichment analysis; MMF, mycophenolate mofetil; PCS, precision cut skin; PPAR, peroxisome proliferator–activated receptor; RNAseq, RNA sequencing; SSc, systemic sclerosis. Color figure can be viewed in the online issue, which is available at http://onlinelibrary.wiley.com/doi/10.1002/art.43151/abstract.

We established precision‐cut skin (PCS) slices from SSc skin biopsies as an ex vivo model system that allows the evaluation of treatment responses directly in the pathophysiologically relevant human target tissue of patients with SSc (Figure 5A). These PCS slices maintain all relevant cellular players and pathophysiologic niches of SSc skin, thereby enabling an ex vivo trial approach for drug testing.^23^, ^24^ Differential gene expression analysis of SSc‐PCS treated with AM1476 (n = 9 patients) compared with donor‐matched PCS treated with the vehicle showed 425 DEGs (*P* ≤ 0.05, |log_2_FC| ≥ 1), 294 of which were up‐regulated and 131 of which were down‐regulated (Figure 5B).

A functional overrepresentation analysis of AM1476‐DEGs demonstrated deregulation of several processes related to tissue remodeling (such as cell adhesion, intermediate filament cytoskeleton organization, intermediate filament‐based process, negative regulation of wound healing, integrin activation, mesenchyme morphogenesis, and negative regulation of myoblast differentiation) and inflammatory responses (such as leukocyte chemotaxis, myeloid leukocyte migration, mononuclear cell migration, granulocyte chemotaxis, neutrophil chemotaxis, and lymphocyte chemotaxis) (Figures 5C). GSEA results highlighted that AM1476 negatively regulated GvHD and induced the peroxisome proliferator–activated receptor signaling pathway,” which is a well‐known antifibrotic signaling pathway (Figure 5D). A direct comparison of AM1476 and mycophenolate mofetil (MMF) revealed that AM1476 regulated more terms related to tissue remodeling than MMF (Figure 5E). MMF is recommended for the treatment of dermal and pulmonary fibrosis in SSc and is the most widely used immunomodulatory treatment used in patients with SSc. Of note, AM1476 and MMF both modulated terms related to various immune cell types, including myeloid leukocytes, granulocytes, and mononuclear cells for AM1476 and T cells, natural killer cells, myeloid leukocytes, and neutrophils for MMF (Figure 5E). These findings suggest that although AM1476 shares some similarities with MMF in regulation of immune responses, it exerts a more pronounced effect on fibrosis‐related terms than MMF in PCS slices of SSc skin.

**Figure 6:**
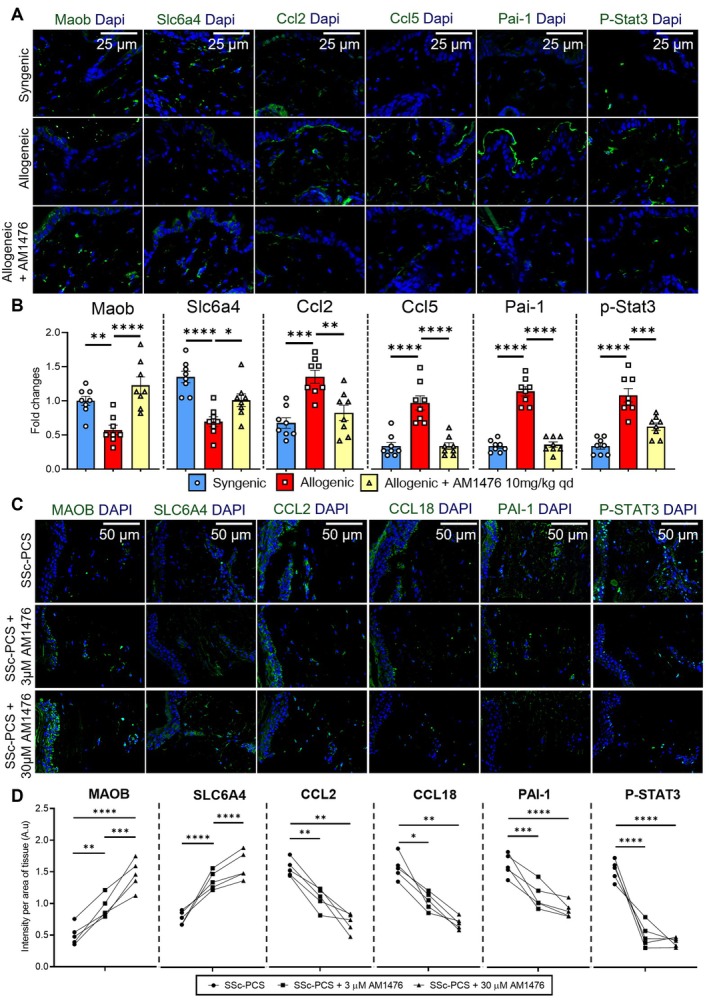
Inhibition of 5‐HT_2B_R by AM1476 modulates the expression of MAOB, SCL6A4, CCL2, CCL5, PAI‐1, and P‐STAT3 in murine cGvHD and PCS slices of SSc skin. (A and B) Murine cGvHD model. (A) Representative images of immunofluorescence staining and (B) quantification of Maob, Slc6a4, Ccl2, CCl5, Pai‐1, and P‐Stat3 in the skin of syngeneically and allogeneically transplanted mice with or without AM1476 treatment. All data are presented as mean ± SEM, with individual values displayed as column plus dots. Differences between the groups were tested for their statistical significance by one‐way analysis of variance with Dunnett's multiple comparison. Adjusted *P* values less than 0.05 were considered significant. Adjusted *P* values are expressed as follows: *0.05 > *P* > 0.01; **0.01 > *P* > 0.001; ***0.001 > *P* > 0.0001; ****P < 0.0001 as compared to the control allogenic group. (C and D) PCS slices of SSc skin. (C) Representative immunofluorescence staining images and (D) quantification of MAOB, SLC6A4, CCL2, CCL18, PAI‐1, and P‐STAT3 in SSc‐PCS with or without AM1476. All data points are presented as individual values displayed as dots. Differences between the groups were tested for their statistical significance by two‐way analysis of variance with Sidak's multiple comparison. Adjusted *P* values less than 0.05 were considered significant. Adjusted *P* values are expressed as follows: *0.05 > *P* > 0.01; **0.01 > *P* > 0.001; ***0.001 > *P* > 0.0001; ****P < 0.0001 as compared to the SSc‐PCS control group. 5‐HT_2B_R, 5‐hydroxytryptamine 2B receptor; cGvHD, chronic graft‐versus‐host disease; PCS, precision cut skin; SSc, systemic sclerosis. Color figure can be viewed in the online issue, which is available at http://onlinelibrary.wiley.com/doi/10.1002/art.43151/abstract.

Integration of RNASeq data from SSc‐PCS and murine cGvHD models, both treated with AM1476, identified up‐regulation of MAOB and SLC6A4 (Supplementary Figure 5). These findings suggest reduced 5‐HT availability in fibrotic tissues treated with AM1476, providing a novel mechanism by which 5‐HT_2B_R inhibition using AM1476 exerts antifibrotic effects. RNASeq data showed consistent up‐regulation of matrix metalloproteinases across both models, suggesting a role in ECM degradation (Supplementary Figure 6A). Additionally, AM1476 down‐regulated WNT5A and WNT10B, two crucial profibrotic cytokines known to promote fibroblast activation and ECM production in tissue fibrosis,^14^, ^25^, ^26^, ^27^, ^28^, ^29^ while up‐regulating WNT antagonists (DKK1, SFRP1/2),^21^, ^29^, ^30^ indicating suppression of WNT signaling (Supplementary Figure 6B). AM1476 also reduced proinflammatory chemokines (eg, CCL2, CCL5, and CCL18)^31^, ^32^, ^33^, ^34^, ^35^ and interleukin‐1 family members^36^, ^37^, ^38^, ^39^ (Supplementary Figure 6C). Finally, AM1476 reduced the levels of P‐STAT3 and PAI‐1, both downstream targets of TGFβ in SSc. Immunofluorescence staining validated the RNASeq findings, showing consistent changes in MAOB and SLC6A4 (markers of 5‐HT metabolism), CCL2, CCL5, and CCL18 (inflammatory chemokines), as well as PAI‐1^40^, ^41^, ^42^ and P‐STAT3 (TGFβ signaling targets)^15^, ^20^, ^23^, ^41^, ^43^, ^44^ (Figure 6A–D and Supplementary Figure 7). These integrated results demonstrate the robust and multifaceted antifibrotic mechanisms of AM1476.

### Safety and tolerability of AM1476 in a phase 1 clinical trial

These findings prompted us to perform a phase 1 clinical trial investigating safety, tolerability, and the pharmacokinetic profile of AM1476 in healthy individuals (ClinicalTrials.gov identifier NCT04691115). AM1476 was well tolerated, most of the adverse events were mild in severity, and no serious adverse events were reported. An excerpt of the summary of adverse events is found in Supplementary Table S5. Following single and multiple oral administration of AM1476 in the fasted state, AM1476 was rapidly absorbed, and steady state was achieved by day two of the multiple‐dose period. The anticipated clinical efficacy exposure was well covered by investigated doses.

## DISCUSSION

We describe herein the pharmacological development of the novel 5‐HT_2B_R inhibitor AM1476 and its effects in preclinical and ex vivo models of SSc. AM1476 overcomes several limitations of previously described 5‐HT_2B_R inhibitors by showing high 5‐HT_2B_R potency with no agonism and with excellent selectivity as well as favorable pharmacokinetic properties. In addition, the low preference of AM1476 to distribute to the brain makes it suitable for chronic treatment, devoid of central nervous system–related side effects.

AM1476 demonstrated potent antifibrotic effects across different preclinical models of SSc. AM1476 reduced fibrotic tissue remodeling in three complementary mouse models of SSc, bleomycin‐induced pulmonary fibrosis, Tsk‐1, and cGvHD, which model different aspects of the pathogenesis of SSc and may resemble different subpopulations of patients with SSc. Bleomycin‐induced pulmonary fibrosis mimics patients with inflammatory, progressive interstitial lung disease in SSc; Tsk‐1 mice resemble patients with noninflammatory, wide‐spread skin disease; and cGvHD mice represent early inflammatory SSc with rapid progression of skin and lung disease. AM1476 reduced myofibroblast differentiation, reduced collagen deposition, and ameliorated dermal and pulmonary fibrosis, respectively, across the different models at well‐tolerated doses. Of note, AM1476 was dosed in a therapeutic manner in all mouse models, with treatment initiation only after fibrosis had already been established. Moreover, we demonstrate therapeutic effects of AM1476 in PCS slices from patients with SSc. PCS slices offer potential to evaluate therapeutic effects of drug candidates in an ex vivo trial approach, with evaluation of drug candidates directly in patient‐derived target tissues with all relevant cellular players and pathogenic tissue niches. AM1476 ameliorated the SSc‐specific gene expression profile and regulated numerous genes and functional processes relevant for the pathogenesis of SSc. Of note, AM1476 regulated more terms related to tissue remodeling in PCS slices than MMF used as an active comparator and representative for the current standard of care. These data highlight antifibrotic effects of AM1476 across different tissues and species.

Our study also reveals that the potent antifibrotic effects of AM1476 might be mediated by a combination of different mechanisms. (1) AM1476 inhibits fibroblast activation in SSc: 5‐HT is released from platelets upon activation at sites of vascular damage in SSc. 5‐HT binds to 5‐HT_2B_R to activate fibroblasts and stimulate collagen release to promote progression of fibrosis. Blockade of 5‐HT_2B_R by AM1476 thus interrupts the link between vascular injury and platelet and fibroblast activation to inhibit progression of tissue fibrosis. (2) AM1476 interferes with the release of inflammatory mediators such as CCL2, CCL5, CCL18, CCL20, IL‐36, and IL‐1RAP. (3) AM1476 inhibits the alternative activation of macrophages in experimental fibrosis. Alternative activation of macrophages is a cardinal feature of SSc and other fibrotic diseases, and AAMs are considered as central cellular players in the pathogenesis of fibrotic tissue remodeling. We demonstrate that AM1476 decreases the number of AAMs in murine cGvHD and reduces functional terms related to macrophage activation in PCS slices of SSc skin. These findings are consistent with previously published results in which 5‐HT was shown to skew macrophage polarization toward AAM through engagement of the 5‐HT_2B_R and 5‐HT_7_ receptor.^45^ (4) AM1476 inhibits innate inflammatory cells. Using RNASeq of PCS slices, we highlight for the first time that inhibition of 5‐HT_2B_R modulates several functional terms related to activation of several innate immune cell subsets of the myeloid lineage. The anti‐inflammatory terms partially overlap with those of MMF, though with more pronounced regulation of myeloid cells and no major effects on T lymphocytes. Although previous studies suggest a direct effect of 5‐HT_2B_R inhibition on macrophages, further studies are required to determine whether the effects of AM1476 on other myeloid cells are mediated via direct effects on these cells or occur indirectly via impaired release of mediators from fibroblasts and AAM activation. (5) We present first evidence that treatment with AM1476 up‐regulates the messenger RNA levels of matrix‐degrading enzymes, providing evidence that 5‐HT_2B_R inhibition may promote ECM degradation. However, these results require confirmation on the protein level and with enzyme activity assays. (6) Consistent with our previous study, we present evidence that AM1476 inhibits TGFβ signaling with down‐regulation of two downstream targets relevant to the pathogenesis of fibrotic tissue remodeling: PAI‐1 and P‐STAT3.^15^, ^20^, ^23^, ^41^, ^43^

Of note, these preclinical findings may have direct translational implications. AM1476 demonstrated good safety and tolerability in a phase 1 clinical trial. Follow‐up trials investigating the efficacy and tolerability of AM1476 in patients with diffuse cutaneous SSc are currently considered.

## AUTHOR CONTRIBUTIONS

All authors contributed to at least one of the following manuscript preparation roles: conceptualization AND/OR methodology, software, investigation, formal analysis, data curation, visualization, and validation AND drafting or reviewing/editing the final draft. As corresponding author, Dr Distler confirms that all authors have provided the final approval of the version to be published and takes responsibility for the affirmations regarding article submission (eg, not under consideration by another journal), the integrity of the data presented, and the statements regarding compliance with institutional review board/Declaration of Helsinki requirements.

## ROLE OF THE STUDY SPONSOR

AnaMar had no role in collection, analysis, or interpretation of the data.

## Supporting information


**Disclosure form**.

**Appendix S1:** Supplementary Information

## Data Availability

All data generated or analyzed during this study are included in this article (and its supplementary files). Additional supporting information or raw data are available from the corresponding author upon request.
