# Supplementary material for: Antifibrotic effects of specific targeting of the 5‐hydroxytryptamine 2B receptor (5‐HT2BR) in murine models and ex vivo models of scleroderma skin
Source: Arthritis Rheumatol. 2025 Apr 17;77(8):1063–76. doi: 10.1002/art.43151 (PMC12311259; doi:10.1002/art.43151)
Supplement: Supplementary file 2 — Appendix S1: Supplementary Information [file ART-77-1063-s002.docx]

**Supplementary materials**

**Specific targeting of the 5-hydroxytryptamine receptor 2B (5HTR2B) demonstrates antifibrotic effects in murine models and ex vivo models of scleroderma skin**

PhD Thuong Trinh-Minh^1,2*^, MSc Cuong Tran-Manh^1,2*^, MD Andrea-Hermina Györfi^1,2^, MSc Nicholas Dickel^3^, BS Christoph Liebel^1,2^, PhD Xiang Zhou^1,2^, PhD Jiucun Wang^4^, PhD Meik Kunz^3^, MSc Helena Arozenius^4^, PhD Lars Pettersson^4^, Sam Lindgren^4^, PhD Christina Wenglén^4*^ and MD Jörg H.W. Distler^1,2*^

* Contributed equally

^1^ Department of Rheumatology University Hospital Düsseldorf, Medical Faculty of Heinrich Heine University, 40225, Düsseldorf, Germany;

^2^ Hiller Research Center, University Hospital Düsseldorf, Medical Faculty of Heinrich Heine University, 40225, Düsseldorf, Germany;

^3^ Chair of Medical Informatics, Friedrich-Alexander University (FAU) of Erlangen-Nürnberg; Erlangen, Germany;

^4^ State Key Laboratory of Genetic Engineering, School of Life Sciences and Human Phenome Institute, Fudan University, Shanghai, China;

^5^AnaMar AB, Stockholm, Sweden

**Supplementary figure**


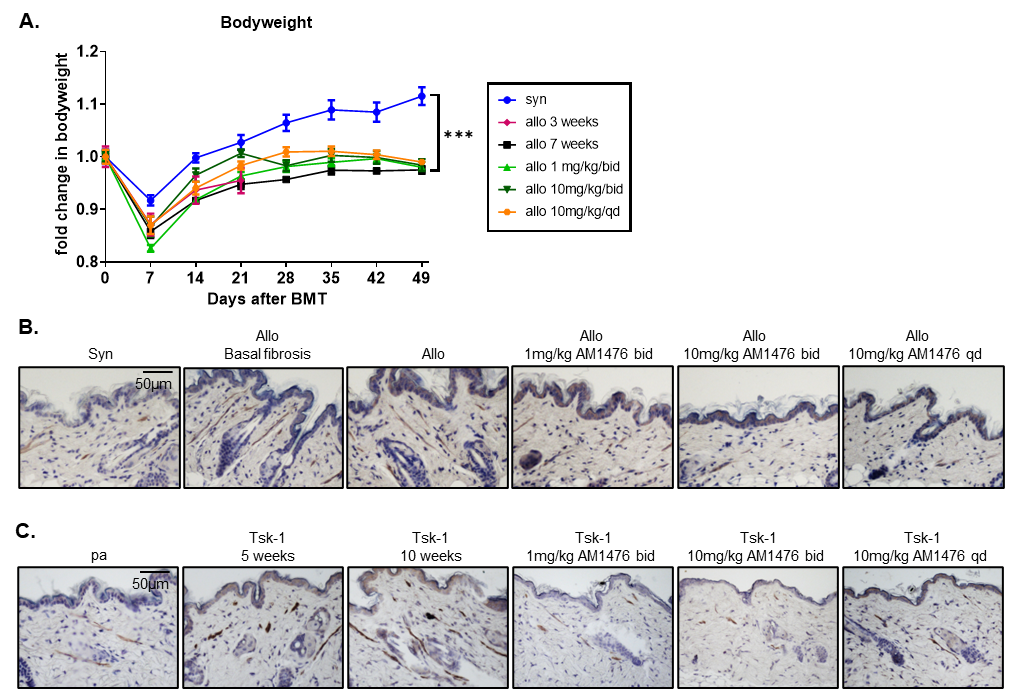


**Supplementary Figure 1.** Effects of AM1476 on body weight. BMT: Bone marrow transplantation. Allo: Allogeneic. Syn: Syngeneic. cGvHD: chronic Graft-versus-Host Disease.

**Supplementary** **Figure 2.** Free plasma concentrations of AM1476 in GvHD mice repeatedly treated with AM1476 for 28 days. The dotted line indicates the IC50 of mouse 5-HT2B receptor antagonistic activity. cGvHD: chronic Graft-versus-Host Disease.

**
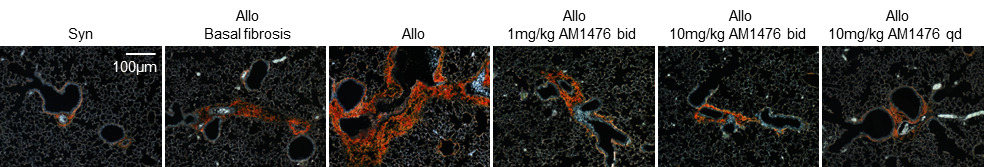
**

**Supplementary Figure 3**. Representative inverted Sirius red staining of cGvHD-induced dermal fibrosis treated with AM1476. Allo: Allogeneic. Syn: Syngeneic. cGvHD: chronic Graft-versus-Host Disease.

**
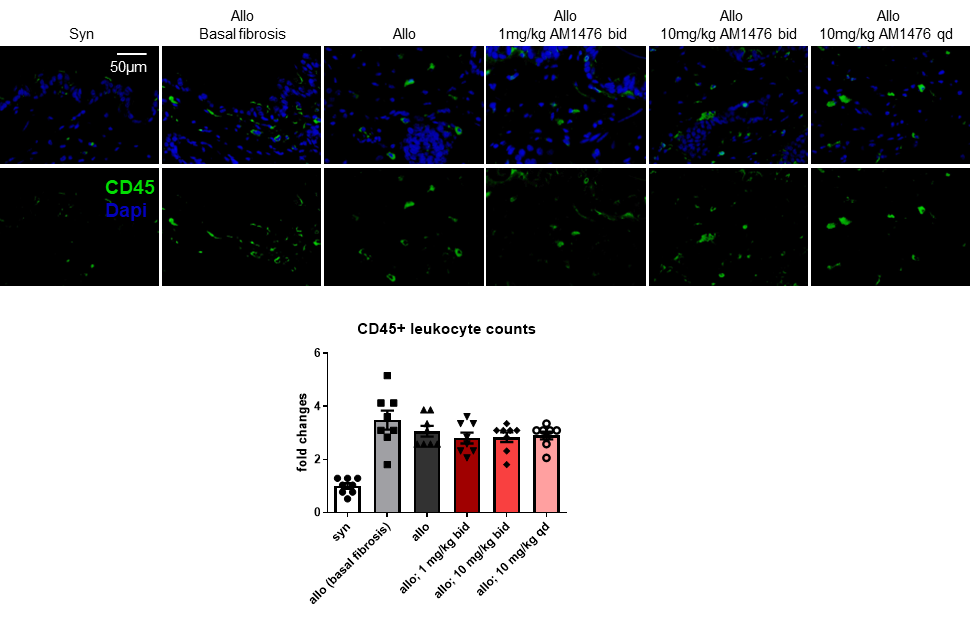
**

**Supplementary Figure 4.** Representative immunofluorescence staining and quantification of CD45^+^ cells of cGvHD-induced dermal fibrosis treated with AM1476. Allo: Allogeneic. Syn: Syngeneic. cGvHD: chronic Graft-versus-Host Disease.


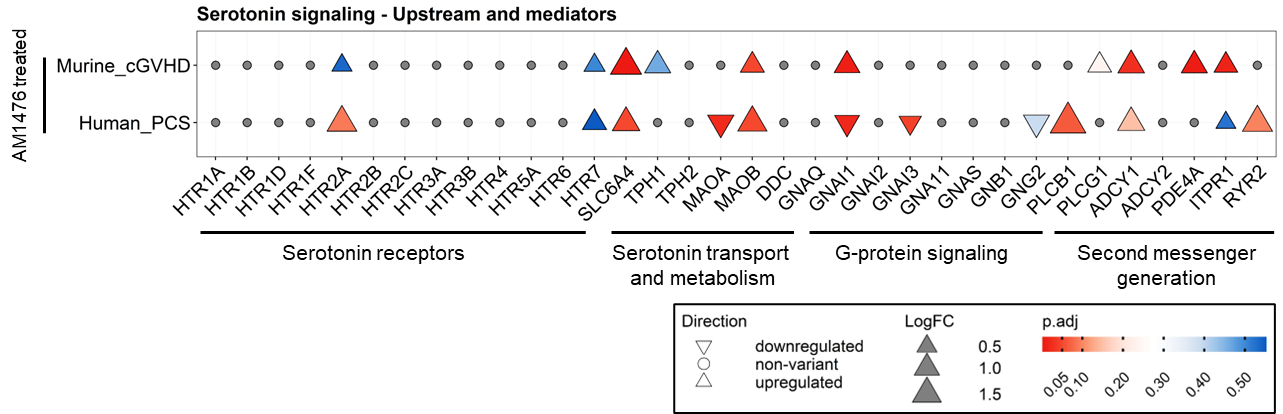


**Supplementary Figure 5.** Changes in core serotonin signaling genes to AM1476 in cGvHD-induced dermal fibrosis and Precision Cut Slices of SSc patients. cGvHD: chronic Graft-versus-Host Disease. PCS: Precision Cut Slice. LogFC: Log 2 of fold change. P.adj: P-adjusted value.

**
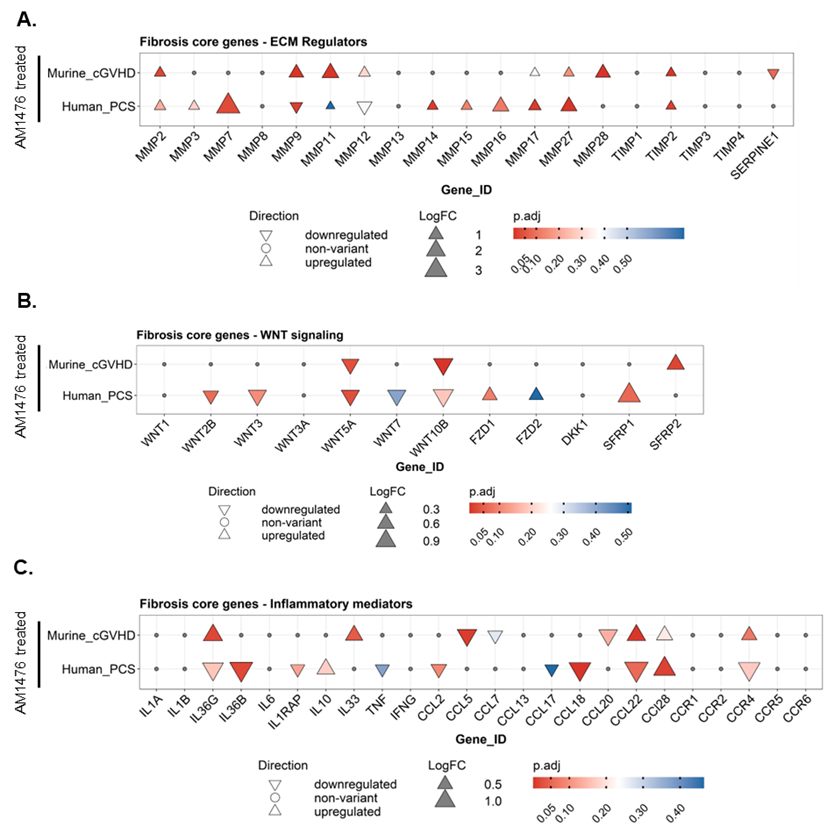
**

**Supplementary Figure 6. Comparative analysis of fibrosis related signaling pathways in AM1476-treated murine cGVHD model and human SSc precision cut skin slices.** The changes of fibrosis related (A) ECM regulators, (B) WNT signaling, and (C) inflammatory cytokines and mediators in skin of murine cGVHD treated with AM1476 and in PCS of SSc patients treated with AM1476. Abbreviations: PCS, Precision Cut Slice; SSc, Systemic sclerosis; cGVHD, chronic Graft-versus-host disease; LogFC, absolute log2 of fold changes; p.adj, adjusted p value, ECM, extracellular matrix.

**
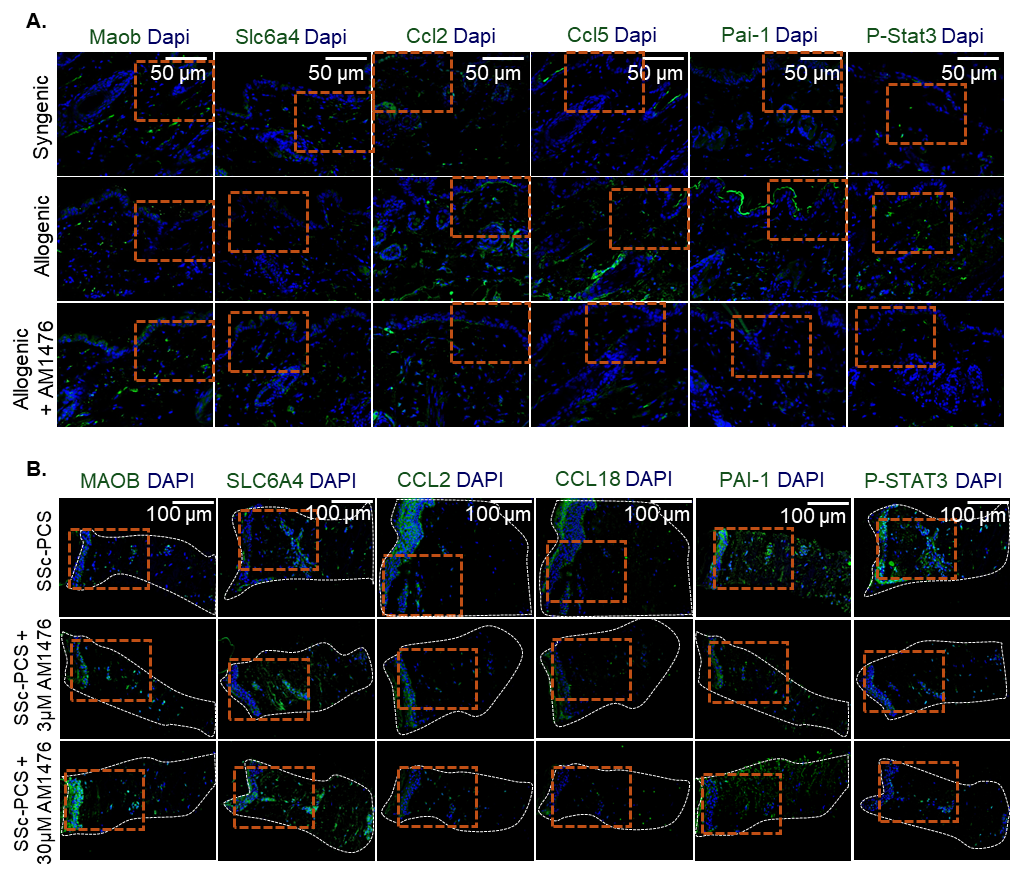
**

**Supplementary Figure 7. Inhibition of 5-HT_2B_ by AM1476 ameliorates inflammatory chemokines and fibrotic markers in murine GvHD skin and Precision cut slices of SSc patients’ skins (SSc-PCS).** (**A**) Low magnification of representative images of immunofluorescence staining of Maob, Slc6a4, Ccl2, CCl5, Pai-1, and P-Stat3 in the skin of syngeneically, allogeneically, or allogeneically transplanted mice treated with AM1476 10mg/kg qd groups. (**B**) Low magnification of representative immunofluorescence staining images of MAOB, SLC6A4, CCL2, CCL18, PAI-1, and p-STAT3 in SSc-PCS treated with AM1476. Orange rectangles indicate the zoom-in areas, which are shown in **Figure 6** of the manuscript.

**Supplementary tables**

**Supplementary Table S1. Demographics and clinical characteristics of SSc patients**

| Parameter | Value |
| --- | --- |
| Total number of patients | 9 |
| Male gender, n (total) | 3 (9) |
| Mean age, years (SD, years) | 60 (7) |
| Mean disease duration (SD) (years) | 4 (4) |
| Mean global mRSS (SD) | 7.89 (3.98) |
| Mean local mRSS (SD) at site of biopsy | 1 (0.47) |
| Progressive skin fibrosis (20% increase of the mRSS within the last year), n (total) | 3 (9) |
| dcSSc, n, (total) | 5 (9) |
| ATA, n, (total) | 4 (9) |
| ACA, n, (total) | 2 (9) |
| ARA, n, (total) | 1 (9) |
| Anti-PM-Scl75/100 antibodies (total) | 2 (9) |
| Inflammatory subtype, n, (total) | 1 (9) |
| SSc-ILD, n, (total) | 5 (9) |
| Mycophenolate mofetil, n, (total) | 3 (9) |
| Nintedanib, n, (total) | 3 (9) |
| Tocilizumab, n, (total) | 1 (9) |
| Rituximab, n, (total) | 0 (9) |
| Methotrexate, n, (total) | 1 (9) |
| Hydroxychloroquine, n, (total) | 2 (9) |

n=number of patients; mRSS= modified Rodnan skin score; dcSSc=diffuse cutaneous systemic sclerosis; ATA=anti-topoisomerase I antibodies; ACA=anti-centromere antibodies; ARA=anti-RNA polymerase III antibodies; SSc-ILD=systemic-sclerosis-associated interstitial lung disease.

**Supplementary Table S2. Comparison of antagonistic activity of AM1476 on human 5-HT_2_ receptors stably expressed by Chinese hamster ovary cells**

| AM1476 | 5-HT_2A_ | 5-HT_2B_ | 5-HT_2C_ |
| --- | --- | --- | --- |
| IC50 | >10 000 nM | 5.8 nM | 3100 nM |

**Supplementary Table S3. Binding (percentage of inhibition) of the 5-HT_2B_R antagonist, AM1476, on a selection of 5-HT receptors**

| **Receptor** | **% Inhibition at 10 (1) µM** | **Assay Cat No (Eurofins Panlabs)** |
| --- | --- | --- |
| Serotonin (5-Hydroxytryptamine) 5-HT_1A_ (hu) | 16 | 271110 |
| Serotonin (5-Hydroxytryptamine) 5-HT_1B_ (hu) | 19 | 271200 |
| Serotonin (5-Hydroxytryptamine) 5-HT_3_ (hu) | 14 | 271910 |
| Serotonin (5-Hydroxytryptamine) 5-HT_4_ (gp) | 74 (34) | 272000 |
| Serotonin (5-Hydroxytryptamine) 5-HT_5A_ (hu) | 70 (10) | 272100 |
| Serotonin (5-Hydroxytryptamine) 5-HT_6_ (hu) | 90 (51) | 272200 |
| Serotonin (5-Hydroxytryptamine) 5-HT_7_ (hu) | 91 (39) | 272320 |

gp=guinea pig; hu=human

**Supplementary Table S4. Functionality of AM1476 on targets, with a potential peripheral mechanism, to which AM1476 displayed a hit in binding assays (defined as ≥50% inhibition at 10 µM)**

| **Targets** | **IC_50_ (M)** | **Comment** | **Assay Cat No (Eurofins Panlabs)** |
| --- | --- | --- | --- |
| Adrenergic α_1A_^h^, IP_1_ (hu) | 4.34E-06 | No significant agonistic activity at 300 µM | 302040 |
| Adrenergic α_1B_^h^, IP_1_ (hu) | 9.53E-06 | No significant agonistic activity at 30 µM | 302060 |
| Adrenergic α_1D_^h^, IP_1_ (hu) | 7.76E-06 | No significant agonistic activity at 30 µM | 302080 |
| Adrenergic β_1_^i^, Adenylyl Cyclase (hu) | 3.25E-05 | No significant agonistic activity at 300 µM | 302230 |
| Adrenergic β_2_^i^, Adenylyl Cyclase (hu) | 1.54E-04 | No significant agonistic activity at 300 µM | 302150 |
| Histamine H_1_^h^, GTPγS Binding (hu) | 2.80E-05 | No significant agonistic activity at 30 µM | 313200 |
| Serotonin 5-HT_6_^h^, Adenylyl Cyclase (hu) | 4.55E-06 | No significant agonistic activity at 30 µM | 318000 |
| Uptake, Dopamine^h^ (hu) | >30E-6 |  | 316010 |
| Uptake, Norepinephrine^h^ (hu) | 3.78E-06 |  | 302000 |
| Sodium Channel, Site 2^h^ (gp) | >30E-6 | No significant agonistic activity at 30 µM | 475010 |

gp=guinea pig; hu=human

**Supplementary Table S5. Excerpt of Summary of Adverse Events by System Organ Class and Preferred Term in Multiple Twice Daily (BID) Doses Cohorts (Complete Summary for Study, see NCT04691115)**

|  |  | **Placebo (N = 6)** | **375 mg AM1476 (BID) (Fasted) (N = 6)** | **500 mg AM1476 (BID) (Fasted) (N = 6)** |
| --- | --- | --- | --- | --- |
|  |  | **no. of subjects (%)** | | |
| Any adverse event* | | 6 (100%) | 2 (33.3%) | 4 (66.7%) |
| Serious adverse event | | 0 | 0 | 0 |
| Nervous system disorders |  | 4 (66.7%) | 2 (33.3%) | 2 (33.3%) |
|  | Headache | 2 (33.3%) | 2 (33.3%) | 1 (16.7%) |
|  | Dizziness | 0 | 1 (16.7%) | 1 (16.7%) |
|  | Depressed level of consciousness | 1 (16.7%) | 0 | 0 |
|  | Presyncope | 1 (16.7%) | 0 | 0 |
|  | Syncope | 1 (16.7%) | 0 | 0 |
| Gastrointestinal disorders |  | 0 | 2 (33.3%) | 1 (16.7%) |
|  | Nausea | 0 | 2 (33.3%) | 1 (16.7%) |
| *An adverse event was defined as an adverse event that started during or after first dosing, or started prior to first dosing and increased in severity after first dosing. Adverse events were coded using the Medical Dictionary of Regulatory Activities. | | | | |
